# Developing a safe culturally competent framework in a multicultural hospital through participatory action research: Study protocol

**DOI:** 10.1371/journal.pone.0329613

**Published:** 2026-02-20

**Authors:** Yasmin El Messoudi-Ahmed Al-lal, Manuel Lillo Crespo

**Affiliations:** 1 Facultad de Ciencias de la Salud, Universidad de Málaga, Málaga, Spain; 2 Nursing Department, Faculty of Health Sciences, University of Alicante, Alicante, Spain; Public Library of Science, UNITED KINGDOM OF GREAT BRITAIN AND NORTHERN IRELAND

## Abstract

**Background:**

Healthcare settings are becoming increasingly multicultural. As a result, cultural factors play a bigger role in patient care. This raises an important question: are health professionals prepared to handle these situations and provide high-quality care?. To answer this, we need to identify the strategies professionals use when caring for culturally diverse patients. We must also determine whether these strategies work, whether they come from training or experience, and how they affect the work environment. The goal of this research protocol is to improve patient safety through culturally competent and culturally congruent care. It focuses on a multicultural hospital located on the border between Spain (Europe) and Morocco (Africa). The project will create a care protocol, a methodology, and a training program based on the organization’s best practices, their experience, and the cultural knowledge of the population usually attended and their feedback.

**Methods:**

The Participatory Action Research (PAR) methodology seems to fit well with the purpose and in the context selected. It will take place in several stages: approach and sensitization, induction, interaction, implementation, and systematization. An estimated sample of 20 healthcare professionals from the regional hospital in Melilla will be invited to participate, though the final number will depend on the saturation of the sample. The analysis will be carried out following Taylor and Bogdan’s criteria. The use of this methodology is intended to have an impact on the participating professionals, since they are part of the process, with their ideas contributing to the elaboration of the results and being complemented with multicultural patients’ views. The duration of the research protocol is estimated between 24–26 months. Approval has already been obtained from the ethics committee.

**Discussion:**

After carrying out all the stages of this research protocol, the next step consists of transferring the evidence and best practices produced into a framework with manuals and protocols that will also be useful in the specific context and will illuminate the type of training recommended for the workforce in such a context.

## 1 Introduction

### 1.1 Background

The increasing number of diverse populations interacting in multicultural environments makes us wonder whether those individuals are ready to do so and, in the case of healthcare providers, if they have been adequately trained to deal with culturally competent and congruent care. If the response is negative, as it happens in this case, our reflection should then focus on how these professionals face such cultural diversity without any specific education, and which tools and good practices they have learned in this context, and work well for them. This leads us to question whether it is appropriate to ask healthcare providers directly about their preparedness in cultural competence when they have never been formally trained to face multicultural situations, as occurs in some published studies. Where, then, does their knowledge come from, and how could such knowledge be useful for others?

This question was already formulated by the main author when planning as an early-stage researcher to explore a multicultural health setting to check what training in cultural competence and transcultural care was available in Spanish-speaking countries. To this end, a preliminary study carried out by the authors revealed a lack of structured and regulated training for nurses in cultural competence at different academic levels in Spain. Although few models exist in Spanish-speaking countries’ undergraduate education aiming at transcultural care training, these are often optional, theoretical, or the content is spread across other topics [1].

This lack of training and consensus on cultural competence in Spanish-speaking contexts means that, when facing multicultural situations, healthcare professionals rely on what they have learned through daily practice. In some cases, they also draw on their belonging to a specific ethnic group or on personal interactions with people from those cultures. As a result, experienced professionals create their own tools, even if these are not based on formal training [2], and daily they transmit such knowledge to others arriving in the context. This experiential learning becomes a resource that fills the gap created by the absence of evidence or structured training. Sometimes, cultural coincidence between professional and patient allows for naturally competent care. The development of all these practices generates that on many occasions professionals are what Dr. Larry Purnell Model name as “unconsciously competent”. This means that even though they may not be aware, because they are used to dealing with different cultures over time, they have developed strategies to provide culturally competent care [3]. Their background may come from patients, colleagues, observation, experience, or, less frequently, additional training they sought themselves. Again, this is experiential learning based on good practices, also known as what works well, despite not being evidence-based.

Having easily and quickly accessible tools that enable healthcare professionals to recall and educate themselves on different cultures is critical [4]. It becomes clear that it is common to leave the ability to deliver cultural care to the personal experience of healthcare professionals. This affects the quality of care, as they are not given the necessary tools but are expected to acquire them on their own, a process that usually takes years. The uncertainty and lack of knowledge that healthcare workers often have are directly linked to a decrease in patient safety [5]. Errors made by newly incorporated staff may arise from various sources. On the one hand, stereotypes, i.e., perceptions held by accepted societies, are usually negative, developed through ignorance of the other culturally diverse person. This problem is accentuated by the slow adaptation on the part of health institutions to the social and cultural changes we face [6]. Another source would be difficulties in communicating with patients due to language barriers. Finally, it could come from the lack of knowledge of cultural aspects that shape how human groups understand health, illness, and the healing process.

Without specific training, it is difficult to avoid prejudice, stereotypes, and ethnocentrism in multicultural contexts, and these situations might potentially incur adverse events and errors that could consequently produce damage in patients [7].

The need to educate and train healthcare providers in the beliefs and ways of life of specific cultures is evident [8]. To achieve this, nursing education programs must be designed according to the needs of a multicultural society. Specific strategies or curriculum interventions should also be incorporated and adapted to each context [9].

### 1.2 Theoretical framework

#### 1.2.1 Seeking equality, inclusion, and justice in healthcare.

Starting from the objective of achieving health care focused on the individual, treating the individual as a biopsychosocial being, and avoiding all forms of inequality in health, thereby reducing the structural inequalities that are intrinsic to healthcare systems, we seek to achieve care based on cultural competence. This is based on different ideologies that have emerged over the years and are worth mentioning. We can name the critical race theory that appeared in the United States, whose objective was based on examining how laws, social structures, or institutions maintained racial inequality, even if there was no individualized racist intention [10]. Other interesting thoughts that we could highlight are both decoloniality and postcolonial thought. Both refer to the same term ‘colonialism’ and, in a way, both seek to deconstruct the legacy of colonialism. However, they have certain differences. Decoloniality thought, which emerged in Latin America and the Caribbean and whose most prominent authors were Aníbal Quijano, Walter Mignolo and María Lugones among others, makes a critique of the coloniality of being, knowledge and power, giving relevance to the separation of Eurocentrism and going against traditionally colonialist structures in global capitalism and race, that is, it tries to overthrow the false universality of Western thought, which takes for granted that its postulates have universal validity, regardless of particular experience [11–13]. On the other hand, postcolonial thought, which emerged in Africa and Asia and whose most prominent authors include Edward Said, Homi Bhabha, and Gayatri Spivak, focuses on deconstructing colonial lines through cultural and linguistic critique to respond to global conflicts [14–16].

Together, decolonial thinking, postcolonialism, and critical race theory enable us to analyze health inequalities as the result of historical power structures and institutional racism, promoting the decolonization of biomedical knowledge, the recognition of diverse cultural practices, and the development of intercultural care models geared towards equity and justice in health [10,11,15].

#### 1.2.2 Transcultural nursing care and cultural competence.

In relation to the search for the cultural competence necessary in professionals to combat these health inequalities, t is well known that ‘culture’ is the union of several factors such as socio-economic status, geographical location, gender, age, ethnicity, or race [1,2,17,18]. UNESCO defines culture as ‘the set of distinctive spiritual, material, intellectual and emotional features that characterize a society or social group, encompassing, in addition to art and literature, ways of life, human rights, value systems, traditions and beliefs’ [3,19]. Concerning health, culture influences how people experience their health and disease process and how they receive medical care and treatment [4,20]. In this regard, several authors have developed models based on cultural competence and transcultural care, such as Larry Purnell with his model of cultural competence and skill acquisition [3]. Health professionals must be trained to act in a culturally competent manner, i.e., they must have the knowledge, attitudes, and behaviors that enable them to work effectively in several multicultural contexts [5,21]. In these contexts, cultural safety becomes particularly relevant, as it positions culture as a central determinant of health and well-being and seeks to ensure that healthcare is delivered in a respectful and non-discriminatory manner. Unlike cultural competence, which focuses on acquiring knowledge, skills, and attitudes to interact effectively with people from different cultures, cultural safety emphasizes the patient’s experience of care, ensuring that interactions do not reproduce power imbalances, discrimination, or cultural harm. Providing culturally safe care requires health professionals to engage in critical reflexivity, meaning they must examine their own cultural identity, positionality, power, values, prejudices, and attitudes, as well as how these factors may influence the patients they care for in the exercise of their professional practice [6–9,22–25]. Regarding cultural safety, reflexivity has several purposes, but probably the most common is the examination of one’s cultural views and values and their influence on the care of people from different cultural backgrounds [10,26].

#### 1.2.3 Participatory Action Research to overcome health inequality and improve health organizations.

To transform this structure, an approach that emphasizes healthcare professionals’ own self-reflection and practical knowledge from the field is essential. Participatory action research (PAR), in which researchers and participants collaborate to generate knowledge and transform practice, was selected as the optimal methodology to achieve this goal. This design can serve as the conceptual model for this study, as it provides a dynamic and collaborative framework for understanding how healthcare professionals construct and transform their practices in multicultural settings from a qualitative perspective. This framework aligns naturally with the concepts of cultural competence and cultural safety, both essential for examining healthcare delivery in culturally diverse environments. Participatory Action Research (PAR) originated with Kurt Lewin in the 1940s, who argued that involving individuals in decision-making enabled theoretical progress and social change to occur simultaneously [27,28]. According to Martínez’s classification [29], the historical development of PAR includes two main strands: a sociological one, represented by Lewin, Sol Tax, and Fals Borda [30], and an educational one, featuring authors such as Freire, Taba, Stenhouse, and Elliott [31]. PAR has since been adopted across fields such as anthropology, social psychology, philosophy, and education, and is understood as an action-oriented research methodology that involves participants in producing knowledge aimed at transforming social reality [32,33]. In multicultural health contexts, this participatory process enables health professionals to become agents of change who actively seek solutions to cultural challenges [34].

Thus, PAR can be included in the critical theory of Guba and Lincoln (2000). In it, research is based on producing critical awareness through the participation of community members [35]. PAR has a series of characteristics, such as considering the participants as social actors having their voice, having as its objective the transformation of the social reality, considering that the problem has its origin in the community, and that it is defined, analyzed, and solved by the participants. On the other hand, it is based on the reality experienced by the members of the community. The dialogue leads to the development of critical awareness in the participants, reinforcing their ability to stimulate themselves to promote change. Finally, it makes participants feel a sense of belonging during the research process [36]. This is fundamental to ensure that research has an impact on health professionals and that it increases the quality of care, so that they become culturally competent.

PAR brings together research, education, and action. Research takes place during the clarification of concerns in the community, as well as helping the members to raise problems. It explores their practices and experiences, two essential parts of healthcare, to give a global, broad, and integral vision of the same reality.

Education takes place by learning from research and investigation; everyone learns from everyone else, sharing their methods and knowledge. In this way, the cultural reality will be known by all members of the community.

The action takes place when critical knowledge originates. The critical conscience arises from the analysis of the situation but also from the commitment to actions to achieve the transformation of the situation [33,36].

There are different PAR models, and almost all of them are quite similar in process and structure since almost all of them are based on and inspired by Lewin’s matrix model [37]. Lewin speaks of cycles of reflective action, each of which consists of several steps: planning, fact-finding, action, reflection, and evaluation [27]. In the field of health sciences, PAR allows a transformation of a concrete situation. It is very useful for health professionals because of the reflective process during the action. This allows them to act actively in the research process, reflect on their practice, develop theories, or select strategies adapted to their context [38]. It should be noted that in PAR, the researcher is also an active subject and contributes to change; therefore, they must begin the research with knowledge about the topic and the field of study, developing empathy with it, and collecting information considering both the discourse and the context [39].

This methodology fits with the objective of the study because, through it, health professionals will be able to reflect on what works for them during the performance of their duties with people from different cultures, as well as problematic situations that may arise from their usual practice. Besides, it will provide us with the tools to collect the necessary information to achieve the ultimate goal of developing a culturally competent and congruent care framework in a multicultural hospital through the creation of protocols and manuals. Its humanistic design grants participation to all those involved, so that they share their knowledge and experiences, learning during the process and acquiring a sense of ownership and commitment to the actions proposed in the practice [40,41]. In addition, it will allow us to create cultural awareness in the professionals during the research, as well as to train them on their ideas, fulfilling one of the PAR foundations that the participants of the research become the agents of change themselves.

For the elaboration of this research, the methodological model proposed by Fals Borda (1987) and Parker (1997) [30,42], intended for social areas immersed in the community, has been used. The authors of these models give instructions to be followed, but do not specify them. This methodology has been used in other works in the health field, such as López et al. (2010), Montero (2016), and Minaya-Freire et al. (2022) [43–45]. The process consists of five phases: Approach and sensitization, induction, interaction, implementation, and systematization.

A wide variety of techniques can be used for data collection as long as they are used rigorously. The researcher and participants work together to establish the best methods of data collection. Qualitative research techniques such as participant observation, interviews, case studies, discussion groups, and participatory methodologies such as the SWOT technique, the participatory assembly, or the nominal group, among others, are recommended [37,46].

It should be noted that qualitative research has a series of characteristics, such as the flexibility of the design that makes it open and changeable throughout the process, the circularity with which, as progress is made throughout the phases, the previous and following phases can be modified, and reflexivity. Reflection allows us to make changes, adjustments, consider the unexpected, and control the research process.

That is, although we can intuit the design of the project and which stages will be developed. Once the project is underway, since it is flexible, there may be circumstances that lead to modifications.

In order to reduce these structural inequalities resulting from a lack of training or evidence-based practices in relation to caring for people from different cultures, this study seeks to develop protocols, training programs, and manuals that will enable professionals to carry out their work based on evidence rather than solely on experience. These kinds of studies are also important to ensure minority or underrepresented voices are included in the investigations.

## 2 Materials and methods

### 2.1 Research questions

The starting conceptual hypothesis comes from the idea that the provision of quality and safe care for populations living in one multicultural context should be assured through specific training and protocols developed for the health professionals attending such populations. Those resources should be informed as well by the professionals’ experiential learning, lessons learned, and good practices acquired over the years, apart from the scientific evidence. To generate the Research Question, we used the PICO framework. For such a purpose, we started with a broad topic, that is to say, we began with a general area of interest, and then we broke it down by using the PICO elements to create the final focused question.

**P:** We identified the specific population (in our case: healthcare professionals).
**I:** We specified the interest/phenomenon (in our case: Their experiences, practices, and strategies in caring for patients from different cultures).
**C:** We identified the context (in our case: A public hospital located in a highly multicultural environment).
**O:** We defined the outcome we would like to generate (in our case: Identification of needs, competencies, and strategies that can be used to develop protocols or manuals to increase the quality of care).

And finally, we formulated the question by combining these elements into a clear, answerable question that would be: How do healthcare professionals at the Hospital Comarcal de Melilla describe their experiences and strategies in caring for patients from different cultures, and which aspects of these experiences can inform the development of protocols or manuals to increase the quality of care?

This structure simplifies the research process and makes it easier to find relevant literature. The study will be conducted following the recommendations of the SRQR (Standards for Reporting Qualitative Research) checklists [47]. Other research questions derived from PICO that have guided the project are:

- Are the health professionals at the HCM culturally competent?
- Is such cultural competence either part of their training or generated by their experience?
- Where does their experience come from?
- What do the health professionals of the HCM know about culturally competent care?
- Are health workers aware of their cultural competence?
- What changes are necessary to improve the quality of culturally competent care?
- How can we assure patient safety in a multicultural context where most health providers have never been trained in cultural competence?

### 2.2 Context and objectives

The Hospital Comarcal de Melilla (HCM) was selected as the study site due to a combination of structural, contextual, and professional factors that make it uniquely suited to the research objectives. As the only public hospital in the Autonomous City of Melilla, HCM is the principal healthcare institution serving the entire local population, which is characterized by a high degree of cultural, ethnic, religious, and linguistic diversity. This centrality positions HCM as an ideal environment for examining healthcare practices in multicultural contexts.

Furthermore, the hospital’s healthcare professionals routinely interact with patients from diverse cultural backgrounds, including Christian, Muslim, Jewish, Hindu, and Roma communities, as well as migrant populations from Morocco and sub-Saharan Africa. This is a particular situation that is only present in this city in Spain. This sustained exposure to cultural heterogeneity provides a natural setting in which culturally responsive professional practices are likely to have developed organically over time [48]. Consequently, HCM offers an unparalleled opportunity to explore how healthcare professionals perceive, interpret, and manage cultural diversity in their daily clinical work.

In addition, the hospital itself employs professionals from a variety of cultural backgrounds, enriching its internal cultural dynamics and fostering a professional environment where intercultural interactions are the norm. This makes HCM not only a site where cultural diversity is encountered externally (through patients) but also internally (within multidisciplinary teams).

Therefore, the general objective of the study is:

- To design a culturally competent and congruent care framework in the HCM that includes a care protocol, methodology, and training based on the center’s best practices, patient safety, and cultural knowledge of the population served.

The specific objectives are:

- To know if the cultural competence of the health professionals at the HCM is part of their training or if it is generated by their experience.
- To describe where the experience of healthcare workers comes from.
- To establish whether health workers are aware of their cultural competence.
- To identify if health professionals of the HCM know about culturally competent care.
- To determine what changes are necessary to improve the quality of cultural care in the HCM.
- To describe how patient safety in a multicultural context, where most of the health providers have never been trained in cultural competence, can be assured.

### 2.3 Study design

The design of this study will be a Participatory Action Research (PAR) with the use of qualitative procedures.

After exploring the available training in cultural competence in Spanish-speaking countries [1] and having concluded the lack of consensus on this topic, we reflected on which methodology could fit with the objectives. This should make it possible to determine what professionals think about this topic and what strategies they apply to face multicultural situations and overcome cross-cultural conflicts. In this way, it would be possible to learn from this and to train other professionals based on the good practices of those who are immersed in these contexts, and to create a continuing education in these issues. The methodology best suited to this objective is Participatory Action Research (PAR). PAR is based on reflection, data collection, as well as initiatives to improve and reduce health inequalities by involving people, who also take action to improve their health [49].

PAR was used to create a working structure to generate important changes in the system and in institutional hierarchies, as well as to address issues related to nursing practice [38].

PAR enables the participation of all parties involved in the process, so it allows them to share their knowledge, experiences, and achieve learning during problem-solving [50]. Participants feel a sense of belonging, ownership, as well as commitment to the actions implemented, as they have participated in their creation and are agents of change [51]. The healthcare workers participating in the study are treated as professionals and experts in their health issues. For this reason, they participate in each phase of the process and can co-design the research itself [52].

Two studies have evidenced the importance of PAR in addressing complex and entrenched nursing issues. This usefulness comes from the great applicability of PAR in nursing both within and outside the Spanish borders. From these studies, it is concluded that PAR contributes to confidence, leadership, and awareness [53,54]. According to these studies, unlike other conventional types of research, this type of methodology makes it possible to apply the results and generate changes in the situation of the participants. This research integrates knowledge into practice. PAR makes it possible to instantly find solutions and make sense of problems in complex clinical settings [54]. So through this methodology, communication can be established with healthcare workers to see what works for them and what does not, and validate this with the population. It is intended to create from this information, training, and protocols. That is to say, to standardize these practices so that other new professionals or those coming from other places can face cultural diversity with tools. It is also intended to make this process useful for the participants themselves to recycle and make them aware of their actions, both good and bad, on culturally diverse patients.

### 2.4 Settings and participants

Non-random purposive sampling will be used, but this does not mean that the quality of the results will be lost. This means that due to the characteristics of the study, it is difficult to obtain random samples. The sampling will be purposive, always considering the criteria of sufficiency and relevance (selecting the participants the most suitable participants who can provide us with the most information according to the inclusion and exclusion criteria mentioned below). The sample will consist of healthcare professionals, specifically nurses, nursing assistants, nursing supervisors, and physicians of the HCM belonging to INGESA (Instituto Nacional de Gestión Sanitaria) in Melilla. The idea is that everyone will participate equally, both in consulting and decision-making. The work unit will not be taken into account, as long as there is direct contact with patients and they meet the inclusion criteria related to work experience listed below. However, a sample of professionals from different cultures will be obtained to enrich the results by providing different perspectives. A balance of perspectives will be ensured, based on years of experience and the departments where they work. For sample recruitment, researchers will use direct contact with participants, as well as notices on notice boards. Informed consent will be used to ensure that participation is voluntary. The sample size will be determined by data saturation, though we will plan to start with approximately 20 participants in the beginning (this is a flexible qualitative design; as the research evolves, this number of participants may be modified in relation to data saturation). The saturation criterion will be taken into account once the information provided by the participants is repeated and does not provide us with anything new, specifically when the information extracted in three successive interviews does not contribute new themes or codes. When this occurs, the criteria of convenience and sufficiency will be achieved, since it will indicate that the sampling has been carried out adequately and the information collected is complete. If, on the other hand, this does not occur, the sample will be increased with more participants.

Although the sample size is not too large, it should be remembered that the objective of the study is not to generalize the results. The aim will be to extract quality information that may be of interest for providing more culturally appropriate care.

It should be noted that the professionals themselves belong to different cultures and can contribute with interesting data from their own experiences. This circumstance may increase the quality of the information as well. Therefore, the focus of attention will be to try to obtain samples belonging to different cultures in addition to the selected work environment. In this way, we will obtain data from different perspectives. It is important to ensure that the informants have the time required to conduct the interview and that they are willing and able to do so.

- **Inclusion criteria**
  - Be a nurse, nurse assistant, or physician.
  - Agree to participate voluntarily.
  - To be active, working in the Hospital Comarcal de Melilla.
  - Have at least 1 year of experience in the organization.
  - Be working or have been working in areas of the hospital where there is direct contact with patients, i.e., hospitalization floors (including the critical care unit), emergency unit, or outpatients.
- **Exclusion criteria**
  - People who do not have enough time to conduct the interview.
  - People who do not have sufficient work experience (1 year at the Hospital Comarcal de Melilla).
  - Not having had any experience dealing with people from different cultures.

### 2.5 Stages of the research

**Fig 1:**
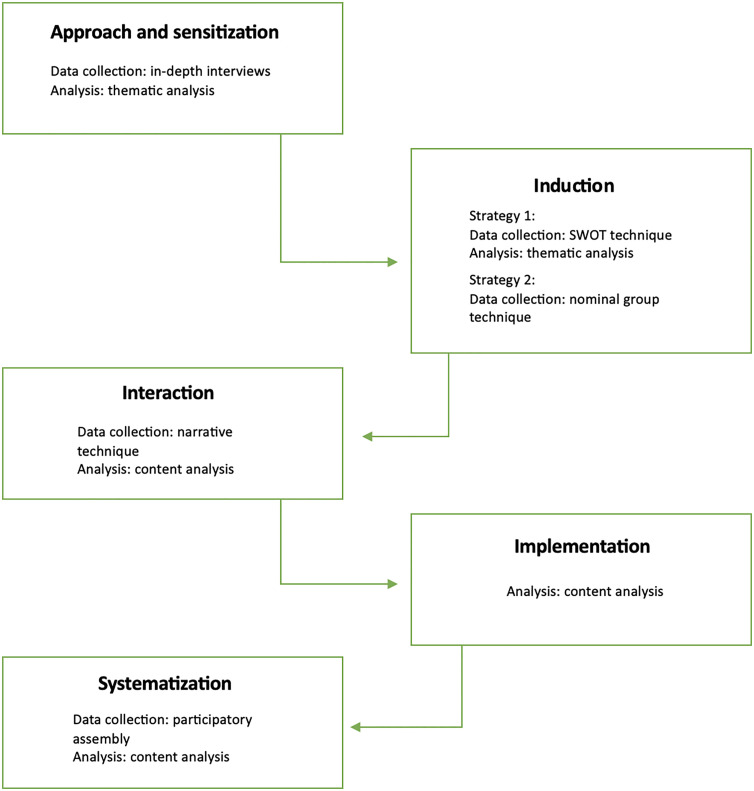
Flow chart of the stages of the PAR.

The study will be carried out in several stages, including data collection and analysis to be performed. The stages of the process are shown in Fig 1.

#### 2.5.1 Stage 1. Approach and sensitization.

The aim is to analyze the healthcare professional’s knowledge of cultural competence, how they carry it out, the different procedures they use, the tools that the hospital has given them, and the tools they have acquired through their own experience. It also establishes whether patient safety criteria are met. In this way, to determine what situation the patients are in and what needs they feel they have.

Action strategy 1: Conceptual and contextual approach to cultural competence. For this purpose, a descriptive qualitative study with a phenomenological approach will be carried out to learn about the experience of health professionals in caring for people from different cultures. “Experience of health professionals when caring for patients with different cultures”. The participants will be HCM health professionals.

Purposive sampling is the number of participants according to the saturation principle, according to representativeness, fulfilling inclusion and exclusion criteria.

Data collection: in-depth interview, semi-structured script, mentioning thematic areas, introducing open questions guided by theory, and aimed at meeting the objectives of the study.

Interviews will be recorded with the consent of the participants. The transcription will be done verbatim. Identifiers will be removed to ensure the protection of participants’ identities. Audio files will be stored securely on a password-protected, encrypted hard drive accessible only to researchers involved in the study. The duration will be between 40 minutes and 1 hour. In a hospital office or wherever possible. Field notes will be collected during the interview.

The analysis proposed by Taylor and Bogdan (1987) will be used [54]. The type of analysis to be carried out will be thematic analysis. Units of meaning are identified and grouped into common themes. Data coding will then take place, and categories and subcategories will be elaborated, trying to capture the meaning of the discourses and taking into account the context in which the data will be collected (ATLAS.ti 9 software will be used as a tool), and finally, the relationship between topics will take place, where the data will be interpreted.

Action strategy 2: outreach to professionals to raise awareness of cultural competence and motivate their participation in the PAR study.

The results of the qualitative phenomenological study will be disseminated through the hospital’s Intranet, where the needs of both patients and professionals will be identified in order to provide culturally competent care (these needs will be identified by the patients and professionals themselves). Other forms of dissemination will be through mailing to both nursing and medical management, posters, leaflets, and social networks, among others.

Participatory assemblies can then be held. With the help of supervisors and/or posters on boards with the date and time of the meeting. Thirty-minute meetings will be held to present the results of the phenomenological study and evidence regarding interventions in the area of cultural competence.

The goal will be to encourage participants to engage in critical discussion as well as explain the purpose of the study and what participation is required.

#### 2.5.2 Stage 2. Induction.

The aim is to identify which factors are related to culturally competent care and to define strategies that enable change in care practice.

Strategy 1: Recognize personal, institutional, and organizational barriers to change and plan strategies for improvement.

Using the SWOT technique, professionals reflect on their reality of care, identify the factors that influence them, and determine strategies for improvement.

- Phase 1: Diagnostic analysis: to determine internal and external factors, both positive and negative, perceived by professionals in order to carry out best practices in the field of culturally competent care. To analyze internal factors, weaknesses and strengths will be identified, and to identify external factors, threats and opportunities will be analyzed. It is intended to answer the question, “What factors influence the implementation of culturally competent best practices?” This stage will start with an individual reflection. Then, there will be a sharing in rounds of participation, with one contribution per turn, until all the conditioning factors that were identified in a matrix of SWOT factors are covered. After that, concepts will be clarified, those with similar meanings will be joined together, and the conditioning factors will be prioritized.
- Phase 2: Strategic analysis: the aim is to establish the conditioning factors identified in the diagnostic analysis in order to draw up a strategy map to promote best practices. Internal and external factors will be cross-referenced to enhance strengths or reduce or eliminate weaknesses, take advantage of opportunities, and reduce or neutralize threats. The objective is to answer the question: What are the action-oriented strategies to promote best practices in the field of culturally competent care?

The phase will be stopped when no new strategies emerge.

The analysis will take place in several phases. In addition to the moderator, two researchers will participate by observing and collecting field notes. At the end of each session, a report will be prepared.

Once the data has been collected in successive pair meetings, a qualitative analysis will be carried out (Taylor & Bogdan, 1987) [55]. Once the analysis is completed, the information will be shared with the participants to check the accuracy of the data.

Strategy 2: Determine the cross-cutting competency profile of professionals to provide more humane, safer, satisfactory, and higher-quality care in culturally competent care.

The nominal group technique will be used.

1st phase: Generation of proposals: each participant will provide the qualities he/she consider: What qualities should the professionals of the HCM have to provide more humane, safe, satisfactory, and higher quality culturally competent care?
2nd phase: Sharing: These are shared with the rest of the group, and more general proposals are generated.
3rd phase: Classification of competencies: Competencies are classified into cognitive, procedural, and attitudinal competencies.
4th phase: prioritization of competencies: importance of the proposals: 1(none), 2(little), 3(some), and 4(a lot).

Descriptive analyses will be carried out to see the profile of the participants.

#### 2.5.3 Stage 3. Interaction.

The aim is to see how reflection can generate alternatives for change.

Strategy 1: Reflect on professional practice to become aware of the capacity for change.

Reading two newspaper articles for participants to make a critical reflection.

The technique to be applied will be narrative. The aim is to link the professionals’ experience, their meanings, and interpretations to their professional practice.

A content analysis (peer review) will be carried out.

#### 2.5.4 Stage 4. Implementation.

Describe the proposed change.

Strategy 1: Determine implementation strategies.

Based on the SWOT matrix, good and bad practices derived from the knowledge of health professionals will be proposed, based on their experience and on scientific evidence.

This will be followed by a content analysis where all proposals will be categorized. The result will be a catalog or manual of good and bad practices that will be sent to the participants for discussion and verification.

Strategy 2: Determine evaluation indicators.

Participants will be given instructions on how to evaluate the implementation strategies. Each participant will propose the indicator he/she consider. The result will be an evaluation procedure that will be sent to the participants for verification.

Strategy 3: Implement the agreed-upon strategies that will drive change.

Small working groups will be formed with a proactive approach.

#### 2.5.5 Stage 5. Systematization.

The incorporation of strategies, the degree of implementation, and the achievements obtained will be examined.

Strategy 1: Evaluate the process of change in health care practice.

A participatory assembly will be held where the results of the implementation will be presented, and positive and negative aspects will be discussed. A report will be generated and sent to the participants for validation. A small advisory panel with patients or family representatives will also be held as a final step to ensure the framework and implementation pathways reflect end-user perspectives.

To ensure the researcher’s reflexivity, meetings will be held every month to analyze the progress and results obtained in each of the PAR phases. Within the research team, the principal investigator has a direct relationship with the regional hospital in Melilla, as she works there. To minimize the possibility of bias, it should be noted that the researchers will carry out daily reflection diaries, which will be shared among the researchers themselves.

It should be noted that the PAR stages are interdependent, so that the results, conclusions, and reflections of the first phase will serve as a starting point for the second phase, where the SWOT and the nominal group will take place. The participants will propose the requested strategies after having discussed and reflected on the results of the first phase. All the perspectives that emerged from the discussions will be taken into account, and if there are conflicting ideas, a middle ground will be sought, and if this is not possible, a simple majority vote among present participants will be taken. The third stage will also begin by reflecting on the results of the preceding stages, to subsequently, through critical reflection, establish a relationship between the experience of the professionals and the meanings and interpretations they make based on the narrative technique.

In phase four, based on the results of the SWOT, good and bad practices will be proposed, and a qualitative analysis will be carried out in which all the proposals will be categorized and returned to the participants for subsequent validation.

The nature of PAR is fundamentally that research participants are an essential part of the project. One of the most important objectives will therefore be for them to actively contribute to the development of the tools that will be generated as a result of the research. In other words, they will be part of the creation of the manuals and protocols. During this process, through reflection and awareness-raising, they will also be formed by the knowledge and experiences they will share. They will also contribute to the establishment of priorities during stage 2, when the SWOT analysis will take place, and also to the elaboration of proposals through the nominal group, as well as to the elaboration of evaluation indicators. Each one will propose one, and the result will be an evaluation procedure that will be sent to the participants for verification. In the participatory assemblies, the degree of implementation will be verified, and the positive and negative aspects will be discussed with them. It is expected that by involving the professionals throughout the whole process in the elaboration of the project, the degree of implementation will be much higher. This is because the professionals will feel an essential part of the project and will develop a sense of ownership.

To summarize, the qualitative techniques that will be used during this study will be interviews, nominal group, SWOT analysis, narrative technique, and participatory assembly. These will be used in the stages described above.

### 2.6 Data analysis

Each PAR stage will have its analysis. Data analysis will be performed by using qualitative thematic analysis (stages 1 and 2) and content analysis (stages 3,4, and 5). Content analysis is a type of text analysis that fragments the text into several units and then codes them according to a pre-established system of categories [56]. Both content analysis and thematic analysis are qualitative methods designed to identify, organize, and interpret meanings within textual data [55]. Both follow a systematic process that will be divided into three main stages according to Taylor and Bogdan’s (1987) proposal [55]. The first stage will be one of discovery in which themes will be identified, and concepts and propositions will be developed. For the analysis, a process of transcribing the data obtained will be carried out at each stage, whether it is the result of interviews, the nominal group technique, the SWOT technique, the narrative technique, or the participatory assembly. Once we have this data, the analysis process will begin, either content-based or thematic, as appropriate. As described above, the objective is to obtain evidence from the analysis that will enable us to develop manuals and protocols. The second stage, once the data have been collected, will be the codification of the data and the understanding of the topic studied. Finally, the stage where the researcher understands the data within the context where they have been collected [55]. While content analysis mainly focuses on describing and classifying information, thematic analysis aims to explore the deeper meanings and patterns expressed by participants.

The analysis will be carried out in several steps [57]:

Step 1: Prepare the data: The information collected from the interviews will be transcribed into written text before starting the analysis. A complete transcription will be made.
Step 2: Define the unit of analysis: understanding the unit of analysis as the basic unit of text to be classified. Coding units will be defined.
Step 3: Develop categories and a coding scheme: Coding schemes can be developed either inductively or deductively. A process of constant comparison will be carried out [58], contrasting the data in terms of their meanings and relationships. Categories in qualitative content analysis do not have to be mutually exclusive, i.e., a text unit can be assigned to more than one category simultaneously. The categories will be elaborated by the persons in charge of carrying out the analysis. A coding manual will be developed that will include category names, definitions, or rules for assigning codes, and examples. This study will be analyzed using a hybrid approach that is both inductive, where codes will emerge from the analysis of the interviews, and deductive, based on pre-established codes from previous literature, in this case from the codes developed by Larry Purnell.
Step 4: Test your coding scheme on a sample of text: A test will be performed on a sample of the data to check the consistency of the coding and validate the coding scheme. If doubts arise, the coding rules as well as the category definitions will be revised. This process will continue until coding consistency is achieved.
Step 5: Code all the text: Once adequate consistency is achieved in the text sample, the coding rules will be used in the rest of the text. However, the consistency review process will be performed repeatedly.
Step 6: Assess your coding consistency: once all the text has been coded, consistency will be checked again to make sure that new codes introduced during the process, possible changes in the coding rules, or even coder fatigue during the process have not led to inconsistencies.
Step 7: Conclude the coded data: In this step, we will make sense of the themes or categories. It is a critical step in the analysis process and depends almost entirely on the analyzer’s reasoning ability.
Step 8: Report your methods and findings. This section will describe in detail the analytical procedures and processes. This will include details of the decisions and practices regarding the coding process established to give reliability to the study. The content analysis will uncover patterns, themes, and categories relevant to the social reality studied.

When presenting the results, a balance will be attempted between description to provide context and interpretation of the data.

Once the results of each stage have been analyzed, they will be sent to the participants in each stage for validation.

The computer software ATLAS.ti 9, will be used for this purpose. This allows qualitative data analysis of texts, graphics, audio, and videos. This form of analysis is useful for analyzing large amounts of verbal data collected through interviews or focus groups and also offers possibilities for quantifying categories.

New data will be collected from health professionals at the HCM and analyzed within the first iteration. According to the results of the previous round, new data will be collected in the following iterations and then analyzed until saturation is achieved. The study will be carried out in different stages and will have a duration of 24–26 months, from the moment the protocol is approved.

To guarantee the transparency of the analytical decisions, as mentioned above, validation of the results obtained in each phase will be carried out with the participants. To ensure trustworthiness and rigor, triangulation of data collection tools will also be used, through the use of interviews, participant observation, nominal group, and narrative techniques. In addition, triangulation will be used in the analysis, as it will be carried out by different researchers, as well as the use of software, in this case, ATLAS.ti 9.

### 2.7 Ethics approval and consent to participate

The project has already received the approval of the Hospital Comarcal de Melilla (S1 Fig) and the approval of the ethics committee of the Hospital Costa del Sol of Málaga (S2 Fig). This is because the Hospital Comarcal does not have its own ethics committee. Participation in the study will be voluntary, and participants will be asked to sign an informed consent form (written) (S3 Fig). Following Spanish Organic Law 15/1999, of 13th December, on the Protection of Personal Data, authorization will be requested from all participants for the dissemination of the results of the project, always guaranteeing their anonymity and confidentiality.

### 2.8 Timeline

To carry out all the stages of the project, it will take 24–26 months. Between 6–7 months to carry out the first stage of approach and sensitization, 6 months for the second stage of Induction, 6–7 months for the third stage of Interaction, and between 7–8 months for the last two stages of Implementation and Systematization.

Recruitment has not yet started, but is expected:

A) Recruitment of participants will start between November and December 2025.
B) Data collection will take place from January to December 2026.
C) Data analysis will take place during 2026.
  Stage 1: From May to July.
  Stage 2: from September to October 2026.
  Stage 3: From November to December 2026.
  Stage 4: from January to March 2027.
  Stage 5: from April to July 2027.
E) Results would be expected by September 2027.
F) Dissemination of results from October to December 2027.

## 3 Discussion

This study will allow us to know whether the professionals of a hospital located in an area characterized by its multiculturalism carry out their professional activity in a culturally competent and congruent way. It will also allow us to know how they have managed to act in this way, whether through training, through their experience, or both, and what cultural practices they have extracted from this experience. In several stages and through different methods of data collection, these objectives will be answered and will also serve as training for the participants. The outcome of this research will allow the development of evidence-based protocols and manuals that will later be used by hospital professionals to act in a culturally competent and congruent manner with the patients from different cultures they serve.

We will ensure the assimilation of the institution by making it and many of its members, some of them managers, aware of the existence and importance of the project. The hospital has explicitly permitted the project to be carried out. Moreover, it deals with a very relevant issue for the city in general, and for HCM in particular, such as transculturality, which means that once we have the results, the assimilation of the project will be easier.

The idea is that once the tools, manuals, and protocols are in place, their usefulness will be evaluated periodically through surveys of professionals, and that in the medium to long term, they can be adapted for use in different hospitals in Spain, in this case, Spain, and even serve as inspiration for hospitals outside this territory.

To disseminate the results, social networks will be used, as well as posters and the hospital’s intranet.

To sum up, unlike other studies, this project is carried out in a hospital located at a border crossing between two countries with different cultures. The location where it will be carried out is a multicultural hospital, accustomed to working with individuals from diverse cultural backgrounds, which is considered a strength of the study. All phases of the PAR will be developed with the aim of increasing patient safety and developing practical tools to improve quality when working with patients from different cultures.

## 4 Limitations

There could be complications in obtaining the sample since the HCM is not a hospital that is accustomed to the participation of professionals in research, so there could be some reluctance. This could affect both their willingness to participate in the study and their capacity and availability. However, given that the principal investigator of the study works at the hospital where the research will take place, and since the sample will be obtained intentionally, it may be easier to get the appropriate sample.

The generalization of the study is limited due to the study methodology, the specific context of the study, as well as the small sample size, and the sample selection method; however, we consider that this information could be useful not only to the center where the study was conducted but also to other centers where there could be patients from similar cultures. The way to extrapolate this information to other hospitals is through the tools developed from the results of this study, i.e., from the manuals and protocols derived from the different stages of the PAR.

It is suggested that in future studies will be carried out with a larger sample, as well as a greater variety of cultures and centers. This study is based on obtaining the perspective of professionals, as it seeks to identify strategies that they have developed over many years of experience working in these contexts. However, in the future, the study could gain further credibility if it included the perspective of patients. This could be an area for further research.

## 5 Conclusion

Achieving culturally competent care is fundamental to ensuring the quality of healthcare professional activity. This increase in the quality of care must begin with awareness and knowledge. Therefore, through this research, we intend to contribute to these two areas. In this way, the healthcare professionals themselves will be the ones who, with the passage of the research stages, will determine areas for improvement, the tools they possess, and the needs of the hospital center, thus contributing to the development of protocols and manuals. These results will serve to ensure that subsequent professional activity will be based on evidence and will no longer be based on experience alone. That is to say, years of experience will no longer be necessary to forge a culturally competent professional work, and the health professionals will have the tools that allow them to perform it from the beginning of their professional activity.

## Supporting information

S1 Fig Authorization of the center where the research will be carried out. (DOCX)

S2 Fig Resolution of the Costa del Sol Hospital Ethics Committee. (DOCX)

S3 Fig Model-informed consent form for study participants. (DOCX)

## Abbreviations

PAR: Participatory Action Research
HCM: Hospital Comarcal de Melilla
