# Supplementary figures and images for: Developing a safe culturally competent framework in a multicultural hospital through participatory action research: Study protocol
Source: PLoS One. 2026 Feb 20;21(2):e0329613. doi: 10.1371/journal.pone.0329613 (PMC12922986; doi:10.1371/journal.pone.0329613)

**S1 Fig. Authorization of the center where the research will be carried out.**


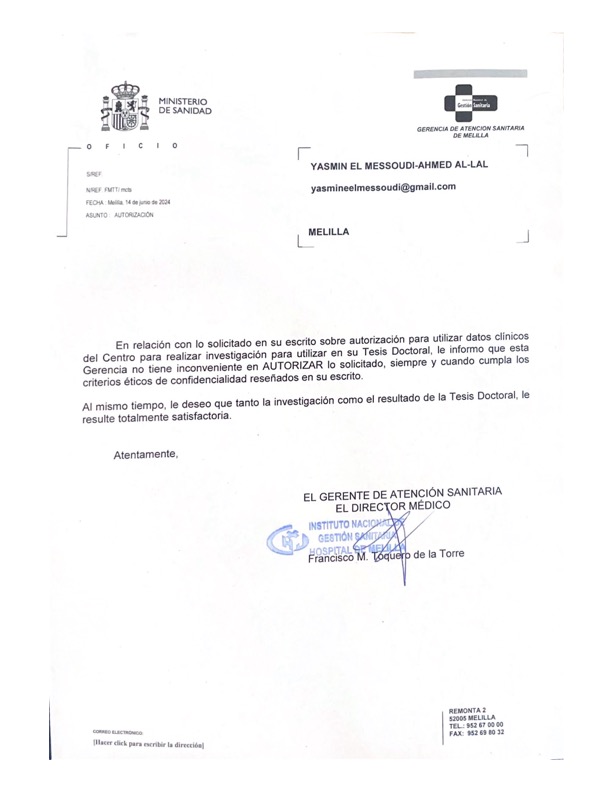

Supplement: S1 Fig — (DOCX) [file pone.0329613.s001.docx]
