# Supplementary material for: Developing a safe culturally competent framework in a multicultural hospital through participatory action research: Study protocol
Source: PLoS One. 2026 Feb 20;21(2):e0329613. doi: 10.1371/journal.pone.0329613 (PMC12922986; doi:10.1371/journal.pone.0329613)
Supplement: S3 Fig — (DOCX) [file pone.0329613.s003.docx]

|  |
| --- |
| **S3 Fig. Model informed consent form for study participants.**  **CONSENTIMIENTO INFORMADO PARA LOS PARTICIPANTES**  **Título**: Desarrollo de un marco seguro y culturalmente competente en un hospital multicultural mediante una investigación-acción participativa  Antes de proceder a la firma de este consentimiento informado, lea atentamente la información que a continuación se le facilita, y realice las preguntas que considere oportunas.  **Justificación y Objetivos del Estudio**:  La finalidad de este estudio es incentivar un cambio en la atención a los pacientes de distintas cultural para lograr otorgar cuidados culturalmente competentes en el Hospital Comarcal de Melilla.  Los objetivos son:  **Objetivo general**   - Desarrollar un marco de atención culturalmente competente, sensible y congruente en el HCM que incluya un protocolo de atención, metodología y formación basado en las buenas prácticas del centro, la seguridad del paciente y el conocimiento cultural de la población atendida.   **Objetivos específicos**   - Saber si la competencia cultural de los profesionales sanitarios del HCM forma parte de su formación o si la genera su experiencia. - Describir de dónde procede la experiencia del personal sanitario. - Establecer si el personal sanitario es consciente de su competencia cultural. - Determinar si los profesionales sanitarios del HCM conocen la atención culturalmente competente. - Determinar qué cambios son necesarios para mejorar la calidad de la atención cultural en el HCM. - Describir cómo se puede garantizar la seguridad de los pacientes en un contexto multicultural en el que la mayoría de los profesionales sanitarios nunca han recibido formación en competencia cultural.   **Diseño y métodos del Estudio:**  El diseño del estudio es una Investigación Acción Participativa. Para llevarla a cabo, se realizará una descripción en profundidad de la situación para después diseñar e implementar estrategias de mejora en los cuidados culturales. Los objetivos se llevarán a cabo utilizando métodos cualitativos. Para la recogida de datos que tendrá lugar de acuerdo a la etapa del estudio dónde nos encontremos utilizaremos diferentes técnicas tales como la entrevista en profundidad, grupos nominal, técnica narrativa, asamblea participativa, técnica DAFO dónde primero se llevará a cabo una reflexión individual y luego se hará una puesta común. Se pueden emplear otras técnicas si el estudio así lo requiriese una vez comenzado el mismo.  Las entrevistas tendrán una duración aproximada de entre 40 y 60 minutos. Si así lo solicitase el participante porque no pudiese acudir al lugar del reunión se facilitarían herramientas tales como ZOOM o Google Meet para tener la reunión de forma telemática.  **Privacidad y Confidencialidad:**  Se garantiza el anonimato de las respuestas obtenidas, la información solo estará en posesión de los investigadores del estudio, si así lo requiere los datos obtenidos podrán ser compartidos con el participante. Los documentos estará protegidos y almacenados, únicamente durante el tiempo necesario hasta la finalización del análisis de la información y la publicación de la misma, momento a partir del cual se eliminarán los documentos.  **Aspectos éticos**: Este estudio de investigación clínica se realizará siguiendo las recomendaciones de la Declaración de Helsinki y la normativa legal vigente en nuestro país en materia de investigación clínica, especialmente la Ley 14/2007, de 3 de julio, de Investigación Biomédica. Este proyecto cuenta además con la conformidad del Comité de Etica “*Hospital Costa del Sol*” y con la autorización del Hospital Comarcal de Melilla.  **Yo:** _______________________________________________________________  DNI/Pasaporte: ____________________________  He leído la hoja informativa que me ha sido entregada  He tenido oportunidad de efectuar preguntas sobre el estudio.  He recibido respuestas satisfactorias.  He recibido suficiente información en relación con el estudio.  He hablado con el Dr./Investigador: Yasmin El Messoudi-Ahmed Al-lal  Entiendo que la participación es voluntaria y que soy libre de participar o no en el estudio.  También he sido informado de forma clara, precisa y suficiente de los siguientes extremos que afectan a los datos personales que se contienen en este consentimiento y en la ficha o expediente que se abra para la investigación:  - Que estos datos serán tratados y custodiados con respeto a mi intimidad y a la vigente normativa de protección de datos (Ley 15/1999 de Protección de Datos de Carácter Personal), por la que debe garantizarse la confidencialidad de los mismos.  - Sobre estos datos me asisten los derechos de acceso, rectificación, cancelación y oposición que podré ejercitar mediante solicitud ante el investigador responsable en la dirección de contacto que figura en este documento.  - Estos datos no podrán ser cedidos sin mi consentimiento expreso y no lo otorgo en este acto.  Entiendo que puedo abandonar el estudio y retirar mi consentimiento:   - Cuando lo desee. - Sin que tenga que dar explicaciones. - Sin que ello afecte a mis cuidados sanitarios   Por ello presto libremente mi conformidad para participar en este proyecto de INVESTIGACIÓN sobre “Experiencia de los profesionales de la salud en el cuidado a personas de diferentes culturas en el Hospital Comarcal de Melilla. Un enfoque desde la investigación acción participativa” hasta que decida lo contrario. Al firmar este consentimiento no renuncio a ninguno de mis derechos. Recibiré una copia de este consentimiento para guardarlo y poder consultarlo en el futuro.  Nombre del paciente o sujeto colaborador:  Firma:  Fecha:  Nombre del investigador: Yasmin El Messoudi-Ahmed Al-lal  DNI: _45308385H_________________________________  Institución/Servicio en el que se realiza el estudio: Hospital Comarcal de Melilla  Dirección de contacto: [yasminelmessoudi@gmail.com](mailto:yasminelmessoudi@gmail.com)  Firma: |
